# Effect of initiating drug treatment on the risk of drug-related poisoning death and acquisitive crime among offending heroin users

**DOI:** 10.1016/j.drugpo.2017.09.017

**Published:** 2018-01

**Authors:** Matthias Pierce, Sheila M. Bird, Matthew Hickman, John Marsden, Graham Dunn, Toby Seddon, Tim Millar

**Affiliations:** a School of Health Sciences, Faculty of Biology, Medicine and Health, University of Manchester, UK; b MRC Biostatistics Unit at University of Cambridge School of Clinical Medicine, Cambridge, UK; c School of Social and Community Medicine, University of Bristol, UK; d Addictions Department, Institute of Psychiatry, Psychology and Neuroscience, King’s College London, UK; e School of Law, University of Manchester, UK

**Keywords:** Heroin users, Treatment, Offending, Acquisitive offending, Drug-related poisoning death, Observational study

## Abstract

**Background:**

A recent Cochrane review of randomised trials identified a lack of evidence for interventions provided to drug-using offenders. We use routine data to address whether contact with treatment services reduces heroin users’ likelihood of a future acquisitive offence or drug-related poisoning (DRP) death.

**Methods:**

Heroin-users were identified from probation assessments and linked to drug-treatment, mortality and offending records. The study cohort was selected to ensure that the subject was not: in prison, in treatment or had recently left treatment. Subjects were classed as initiators if they attended a triage appointment within two weeks of their assessment; non-initiators otherwise. Initiator and non-initiators were compared over a maximum of one year, with respect to their risk of recorded acquisitive offence or DRP-death. Balance was sought using propensity score matching and missing data were accounted for using multiple imputation.

**Results:**

Nine percent of assessments identified for analysis were classed as initiators. Accounting for observed confounding and missing data, there was a reduction in DRPs associated with initiator assessments, however there was uncertainty around this estimate such that a null-effect could not be ruled out (HR: 0.42, 95% CI 0.17–1.04). There was no evidence of a decrease in the recidivism risk, in fact the analysis showed a small increase (HR: 1.10, 95% CI 1.02–1.18).

**Conclusion:**

For heroin-using offenders, initial contact with treatment services does not appear to reduce the likelihood of a future acquisitive offence.

## Introduction

Endemic heroin use is associated with significant public health and social problems (UN Office On Drugs and Crime, 2010): in particular, high rates of mortality (Degenhardt et al., 2013; Pierce, Bird, Hickman, & Millar, 2015) and acquisitive offending (Bennett, Holloway, & Farrington, 2008; Pierce, Hayhurst et al., 2015). In the UK, structured addiction treatment is commissioned with the aim of reducing users’ dependence on illicit drugs and minimizing the harms associated with these drugs, including premature death and offending (Home Office, 2010). The front-line intervention offered for heroin dependence is opioid substitution therapy (OST) with methadone or buprenorphine (National Treatment Agency for Substance Misuse, 2006). Psychological support is also available but recommended only as an adjunct to OST (National Treatment Agency for Substance Misuse, 2006). In the UK, the treatment pathway for patients with heroin dependence is determined during a triage appointment with a drugs key-worker (National Treatment Agency for Substance Misuse, 2006).

In medical and social research, randomised controlled trials (RCT’s) are considered optimal for assessing the effectiveness of an intervention (Campbell, Stanley, & Gage, 1966). However, RCTs and meta-analysis of interventions provided for heroin users have been underpowered to detect changes in mortality or offending and usually focus on intermediate outcomes such as reduced illicit opioid use and treatment retention (Amato et al., 2013; Amato, Minozzi, Davoli, & Vecchi, 2011a; Amato, Minozzi, Davoli, & Vecchi, 2011b; Mattick, Breen, Kimber, & Davoli, 2009; Mattick, Breen, Kimber, & Davoli, 2014). For example, a recent meta-analysis of RCTs for OST offered for heroin-using offenders was unable to detect an effect on future arrests (1 study, 62 subjects, RR: 0.60, 95% CI: 0.32–1.14) or incarceration (3 studies, 472 subjects, RR: 0.77, 95% CI: 0.36–1.64) (Perry et al., 2015).

Cohorts identified from routinely collected data can provide the necessary power to investigate rarer outcomes (Bird, 2008). Many studies of addiction treatment aim to quantify the effect of being treated by contrasting periods in and out of treatment. However, this will be a biased comparison if there are non-random reasons for why patients leave treatment which are related to the outcome under consideration. To account for this confounding bias, confounding variables should be measured over follow-up; however, such information is rarely available or incomplete. This problem can be avoided by analysing subjects according to initial treatment status — something closer to the intention to treat principle routinely used in randomised controlled trials. Treatment and control subjects can then be balanced prior to follow-up, using propensity score methods.

This study used a large, observational, record-linkage dataset from England, to analyse the effect of initiating drug-treatment on subsequent offending and mortality. We focus on the effect of initiating treatment, ignoring the fact that many who begin treatment may drop-out early. Therefore, our study aims to quantify the impact of a policy where everybody with heroin dependence attends a triage appointment. The study is designed to best emulate what would have occurred during an RCT — an approach that has been recommended in pioneering work from other areas of clinical research (Danaei, Rodriguez, Cantero, Logan, & Hernan, 2013; Hernan et al., 2008, Toh and Manson, 2013).

We use this design to investigate two hypotheses: for heroin users identified in the criminal justice system, does initiating contact with treatment services reduce the risk of: (a) a future drug-related poisoning death and (b) a recorded acquisitive offence.

## Methods

A cohort of heroin users was identified from probation (offender management) assessments, using inclusion and exclusion criteria. Following an eligible probation assessment, if subjects were recorded in treatment data as attending a triage appointment within two weeks they were classed as an *initiator*; otherwise they were classed as a *non-initiator*. Outcome events were defined as a drug-related poisoning (DRP) death or a day when the subject committed a recorded acquisitive offence over a maximum of one-year. Time-to-outcome was compared between initiators and non-initiators, irrespective of future treatment status. Balance between initiators and non-initiators was sought by matching on propensity scores calculated using an extensive set of baseline covariates available from probation assessment and historical offending records.

### Datasets

Data were extracted from the Drug Data Warehouse — a collection of case-linked national datasets on substance users in England, covering the period 1st April 2005 to 31st March 2009 (Millar et al., 2012).

The analysis cohort was identified from probation assessments recorded on the Offender Assessment System (OASys) database. OASys contains information from a structured interview between offender and probation officer with the aim of assessing an offender’s recidivism risk and to identify particular needs (National Probation Services, 2003). This assessment can form part of a pre-sentence report, to aid the judge’s sentencing decision, or can be used to help probation services manage offenders post-sentence, for example after release from prison on licence (i.e. serving the remainder of a sentence in the community, under regular supervision by probation services).

Treatment data were obtained from the National Drug Treatment Monitoring System (NDTMS). NDTMS collects data on contact between substance-use disorder patients and structured treatment delivered by National Health Service and third-sector providers, which together account for almost all such provision in England. When a substance-use disorder patient initially contacts treatment services they undergo a triage appointment with a key-worker. The aim of this appointment is to assess the patient’s needs and determine the most appropriate treatment. After this appointment, clients may be offered treatment within the assessing treatment agency, or onward referral to another service.

Details of sanctioned offending were determined through Police National Computer (PNC) records, for all offences that occurred since the age of ten, and resulted in a conviction, caution, warning or reprimand. A death occurring over follow-up was established from national mortality records.

Linkage was done based on a minimal identifier (initials, date of birth and gender). Additionally, criminal-justice system databases included an individually unique CJS identifier. Due to data release requirements, instances where more than one CJS identifier linked to a single minimal identifier were removed because this provided evidence that multiple subjects shared the latter details. This affected 33.6% of assessments in OASys and these were dropped from the analysis. Identifiers were fully anonymised prior to their release to the study team.

### Inclusion/exclusion criteria

**Fig. 1:**
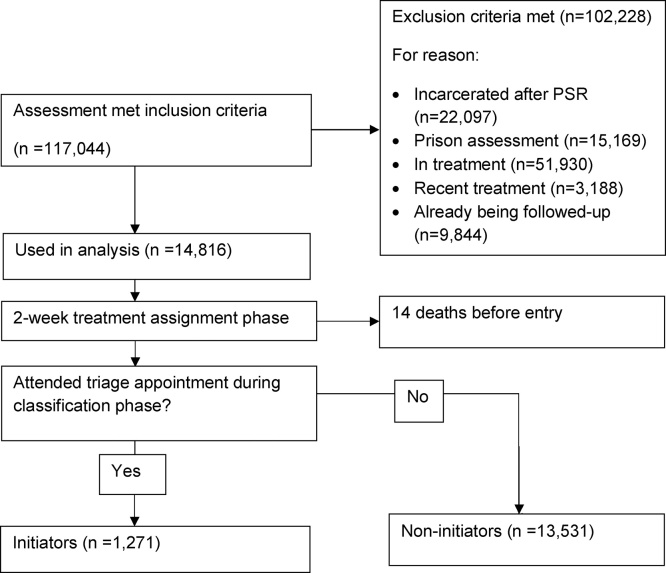
Flow-chart of selection of probation assessments into the analysis cohort.

Probation records were included in the analysis cohort provided the interviewed subject: was assessed between April 1 2005 and March 1 2009; reported weekly or more frequent use of heroin (by any route of administration); was aged 18–64 years. After resulted in 117,044 assessments (see Fig. 1).

A priori criteria were established so that, in turn, probation records were excluded from the study if:

(i) The assessment was for a pre-sentence report which was associated with a subsequent prison sentence (n = 22,097)
(ii) The assessment was post-sentence but carried out in prison (n = 15,169)
(iii) The subject was already in treatment at the time of their probation assessment (n = 51,930)
(iv) The subject had left treatment in the four weeks prior to their probation assessment (n = 3188)
(v) The subject was already in the study at the time of assessment via a previous assessment (n = 9844)

The rationale for these exclusion criteria were: (i and ii) time incarcerated will be associated with lower DRPs, due to restricted access to drugs, and (naturally) offending risk and our data did not include date of prison release; (iii) the focus of the analysis is on the effect of initiating treatment, not on the effect of having been in treatment for some time; (iv) the four weeks following treatment cessation are known to confer elevated overdose risk (Pierce, Bird, et al., 2016) and so this cut-off was chosen as a wash-out period, prior to subsequent treatment; (v) was to ensure that no person could be entered into the analysis if they were already under observation.

### Exposure definition

We designated a two-week *classification phase* that followed an eligible probation assessment. During this period, if the subject attended a triage appointment for opioid misuse treatment, as recorded in NDTMS data, they were classified as an *initiator* and the remainder who did not have any treatment contact were defined as a *non-initiator*. This two week cut-off was chosen by members of the research team, prior to analysis, as it was considered that a shorter period missed too many initiators, and a longer period would mean that the variables collected at the assessment would no longer be relevant.

Fourteen deaths occurred during the classification phase and these were excluded from analysis. Follow-up began after this period and ended on the earliest date of: the outcome under consideration, the end of data collection (31st March 2009), death from any cause, or one year after baseline assessment. The resulting analysis dataset consisted of 14,802 assessments (13,204 individuals).

### Outcome definitions

An acquisitive-offence event was defined using the offence date, recorded in the PNC, for sanctioned offences with an offence code identifying the crime categories: theft, fraud and forgery, burglary, robbery, drug supply (including intent to supply) and prostitution. The PNC does not differentiate between drug-supply for material gain and ‘social supply’, whereby drugs are shared between individuals. Inclusion of drug-supply in the definition of ‘acquisitive offences’ is intended to increase its sensitivity, at the cost of some specificity. A fatal drug-related poisoning (DRP) was defined according to the UK harmonised definition (Office for National Statistics, 2016) using the ICD-10 code (World Health Organization, 2010) for the underlying cause of death.

### Propensity scores

Propensity scores give the probability of a subject being an *initiator*, conditional on a set of baseline covariates. If the model used to predict propensity scores is correctly specified, then the covariates will be evenly distributed between subjects with the same propensity score. It follows that if all possible confounders are used to predict the propensity score – that is all factors are identified which can influence both self-classification and outcome – then the contrast between initiator and non-initiators with the same propensity score will be unconfounded (Rosenbaum & Rubin, 1985).

Propensity scores were calculated using a logistic regression model which included variables available in the OASys system or calculated from historical PNC records. For ease of presentation, these covariates were grouped into eight types: demographic; relating to the assessment; relating to the current offence; drug use; alcohol use; employment and housing; mental and physical wellbeing; and offending history. All identified covariates were used to calculate the propensity scores. A description of each covariate is available in Appendix A in Supplementary material.

### Propensity score matching and assessing balance

Once propensity scores were calculated, the variable matching approach recommended in Rassen et al. (2012) was adopted, with up to *five non-initiators* selected for each *initiator.* The matching algorithm is described in detail in Appendix B in Supplementary material. The calliper distance was set as 20% of the standard deviation of the logit of the propensity scores (Austin, 2011a).

Balance for each covariate, both prior to and post matching, was assessed using its standardised distance between *initiator* and *non-initiators*. This was calculated for each *initiator* assessment’s worst match (i.e. the match with the greatest propensity score distance) to get a conservative measure of balance. The resulting model was considered poorly balanced if the standardised difference between pairs was >10% of the standard deviation (Austin, 2011b).

### Analysis model

Proportional hazard models were fitted, contrasting the time to event (acquisitive crime or DRP) between i*nitiators* and *non-initiators.* Subjects entered the risk set after the classification phase (i.e. two week’s post-assessment). Multiple assessments belonging to the same individual were accounted for by adjusting the standard errors using the Huber-White sandwich estimator.

The same propensity score matched groups were used for the analyses of both outcomes. The matched sample was analysed by stratifying the Cox model on each matched group. *Non-initiators* were given weights equal to the inverse of the number of *non-initiators* in that matched group. For example, within a group with a matching ratio of 1:4 each *non-initiator* would be given ¼ weights and the *initiator* was given a weight of one. The Schoenfeld residuals were examined for departures from proportional hazards.

### Missing data

Thirty percent (4480/14,802) of assessments in the analysis dataset had missing data for at least one of the baseline covariates used to calculate the propensity score. Initially those with missing data were excluded from the matched analysis. To account for potential selection bias in this approach, those with missing information were incorporated into the analysis using multiple imputation (ICE command, Stata 13) (Royston & White, 2011). Missing data distributions were calculated using all variables in Appendix A in Supplementary material, plus indicators for being an i*nitiator* and whether the subject had a death or an offence over follow-up. Ten imputed datasets were constructed and a propensity score model was fitted to each. The multiple imputation propensity score was taken as the mean of these and the matching process was repeated (Hill, 2004).

### Sensitivity analysis

A planned sensitivity analysis was conducted to test the robustness of the results to one element of the study design: the definition of an *initiator*. This was performed by changing the length of the classification phase from two weeks to one week and then four weeks.

## Results

There were 14,802 assessments identified for analysis and 12,948 person years of follow-up (Table 1). Nine percent of assessments (1271) were classed as *initiators*.

**Table 1:** Summary of key statistics, initiators and non-initiators.

Statistic	Initiators		Non-initiators	
	N	%*	N	%*
Number of people	1,255		11,949	
Person years	1,104		11,844	
Assessments	1,271		13,531	

Assessments with missing data for any variable	304	24	4,176	31

Assessments in 1:5** matched sample	908	71	3,790	28
Person years	780		3,247	

Assessments in 1:5** matched sample after multiple imputation	1,201	94	5,047	37
Person years	1,044		4,408	

* Percentage of all assessments.

** As the algorithm used to calculate the matched groups allowed initiator to have up-to (not exactly) five matches within the pre-specified calliper bounds the final ratio was not 1:5 (actual ratio 1:4.3).

Prior to matching, the standardised difference between *initiators* and *non-initiators* was small for many covariates (Table 2). For example, *initiators* had a similar proportion of women and a similar mean age to *non-initiators*. There were differences with respect to the purpose of the baseline assessment: initiators were more likely to have been assessed as the result of a pre-sentence report, rather than a sentence review or start of their release on licence. *Initiators* were also more likely to be daily users of heroin and less likely to have declared alcohol use as a significant problem.

**Table 2:** Comparison between initiators and non-initiators on baseline covariates, classified within 8 groups, prior to and post propensity score matching.

Variable	Initiators N = 1271		Non-initiators N = 13,531		Standardised difference (%)	
	N/mean	%/±SD	N/mean	%/±SD	All	Matched
Group 1: demographic variables						
Gender						
Male	955	75	10,422	77	−4.4	−2.1
Female	316	25	3109	23		
Mean age	31.1	±7.0	31.7	±7.3	−8.1	−3.4
Ethnicity						
White	1084	85	11,246	83	4.6	−0.8
Non-white	106	8	1284	9		
Missing	81	6	1001	7		
Region						
Eastern	87	7	1119	8	−5.4	0.0
East Midlands	185	15	1465	11	11.2	−3.2
London	85	7	1246	9	−9.3	2.8
North East	70	6	677	5	2.3	5.1
North West	264	21	2676	20	2.5	−1.9
South East	154	12	1728	13	−2.0	1.4
South West	84	7	1031	8	−3.9	−0.5
West Midlands	166	13	1699	13	1.5	1.0
Yorkshire& Humber	176	14	1890	14	−0.3	−1.8

Group 2: relating to the assessment						
Purpose of assessment						
Pre-sentence report	456	36	2695	20	36.2	−1.1
Start community sent	366	29	3736	28	2.6	4.8
Sentence review	119	9	2264	17	−22.0	0.0
Start licence	135	11	2361	17	−19.8	−1.5
End sentence	195	15	2475	18	−7.9	−3.3
Sentence tied to the assessment						
Community	596	47	5225	39	15.4	−2.0
Custody	89	7	1556	12	−17.7	−1.3
Suspended	159	13	1448	11	4.7	−4.1
Other	198	16	1993	15	1.0	3.0
Community punishment	111	9	1641	12	−13.1	6.0
Missing	118	9	1668	12		
Mean assessment no.	1.11	±0.33	1.13	±0.36	−5.0	−3.9
Assessment financial year						
2005–2006	355	28	4213	31	−7.0	4.3
2006–2007	287	23	3073	23	−0.3	−0.5
2007–2008	308	24	3065	23	3.7	−1.3
2008–2009	321	25	3180	24	4.1	−2.5

Group 3: Offending which led to assessment						
Offence which resulted in the probation assessment						
Violence/sexual	103	8	1273	9	−4.7	0.0
Serious acquisitive	210	17	2445	18	−4.2	−1.5
Non-serious acq.	618	49	5991	44	8.6	−1.1
Drugs offences	155	12	1747	13	−2.3	1.0
Other	176	14	1945	14	−1.6	2.2
Missing	9	1	130	1		
Are current offence(s) an escalation in seriousness from previous offending?						
Yes	234	18	2823	21	−6.5	0.0
No	1007	79	10,342	76		
Missing	30	2	366	3		
Are current offence(s) an established pattern of similar offending?						
Yes	1044	82	10,840	80	4.9	−0.9
No	217	17	2560	19		
Missing	10	1	131	1		

Group 4: Drug use/problems						
Was there evidence of motivation due to addictions/perceived needs?						
Yes	887	70	9055	67	5.0	−4.2
No	363	29	4136	31		
Missing	21	2	340	3		
Did drugs act as a disinhibitor?						
Yes	862	68	8774	65	5.8	−6.5
No	395	31	4553	34		
Missing	14	1	204	2		
Daily use of heroin?						
Yes	1009	79	9720	72	17.7	1.9
No	262	21	3811	28		
Currently injects heroin						
Yes	516	41	4896	36	9.1	−5.1
No	755	59	8635	64		
Previously (but not currently) injected heroin						
Yes	279	22	3076	23	−1.9	−0.3
No	992	78	10,455	77		
Other opiates or (non-prescribed) methadone						
Yes	136	11	1543	11	−2.2	0.0
No	1135	89	11,988	89		
Crack use						
No	765	60	7627	56	7.8	7.9
Occasionally	133	10	1286	10	3.2	−4.1
Frequently	373	29	4618	34	−10.3	−5.8
Previously used crack						
Yes	350	28	3338	25	6.5	2.0
No	921	72	10,193	75		
Cocaine/amphetamine use						
Yes	71	6	851	6	−3.0	−3.4
No	1200	94	12,680	94		
Benzodiazepine use						
No	1157	91	12,425	92	−2.8	1.9
Occasionally	40	3	316	2	5.0	−1.3
Frequently	74	6	790	6	−0.1	−1.3
Other drug used?						
Yes	60	5	692	5	−1.8	−4.3
No	1211	95	12,839	95		
A current injector of a drug aside from heroin?						
Yes	410	32	4412	33	−0.7	−3.7
No	861	68	9119	67		
Violent behaviour related to drug use?						
Yes	263	21	3129	23	−6.1	−6.3
No	998	79	10,258	76		
Missing	10	1	144	1		
Motivated to tackle drug misuse?						
Yes	368	29	3431	25	8.1	2.7
Somewhat	754	59	7859	58	2.6	−1.6
No	145	11	2201	16	−14.1	−1.3
Missing	4		40			
Drug use and obtaining drugs a major activity/occupation?						
No	140	11	1721	13	−5.3	1.1
Somewhat	470	37	4795	35	3.1	1.8
Significantly	659	52	6978	52	0.4	−2.4
Missing	2		37			
Drug misuse issues linked to risk of serious harm, risks to the individual and other risks?						
Yes	814	64	8274	61	−6.2	−5.1
No	445	35	5144	38		
Missing	12	1	113	1		
Drug misuse issues linked to offending behaviour?						
Yes	1213	95	12,829	95	3.7	−4.7
No	49	4	623	5		
Missing	9	1	79	1		

Group 5: Alcohol						
Did alcohol act as a disinhibitor?						
Yes	178	14	2337	17	−9.8	0.9
No	1046	82	10,530	78		
Missing	47	4	664	5		
Is current alcohol use a problem?						
No	966	76	9687	72	10.1	0.5
Somewhat	166	13	1829	14	−1.4	−1.6
Significantly	128	10	1886	14	−12.0	1.1
Missing	11	1	129	1		
Binge drinking or excessive use of alcohol in the last 6 months?						
Yes	280	22	3415	25	−7.7	2.4
No	980	77	9968	74		
Missing	11	1	148	1		
Frequency and level of alcohol in the past						
No problems	897	71	9155	68	6.1	−1.5
Some problems	125	10	1536	11	−5.0	0.0
Significant problems	238	19	2694	20	−3.1	1.7
Missing	11	1	146	1		
Motivated to tackle alcohol misuse?						
No problems	1030	81	10,449	77	9.3	−3.2
Some problems	188	15	2260	17	−5.4	2.2
Significant problems	41	3	669	5	−8.8	2.5
Missing	12	1	153	1		
Alcohol misuse linked to offending behaviour						
Yes	326	26	3884	29	−7.3	3.5
No	935	74	9467	70		
Missing	10	1	180	1		

Group 6: Employment/housing						
Unstable accommodation						
Yes	755	59	7504	55	7.7	0.9
No	506	40	5882	43		
Missing	10	1	145	1		
Unemployed						
Yes	1166	92	12,287	91	1.6	0.4
No	94	7	1052	8		
Missing	11	1	192	1		
In receipt of benefits?						
Yes	1050	83	10,891	80	4.5	−1.2
No	198	16	2320	17		
Missing	23	2	320	2		
Financial issues linked to offending behaviour						
Yes	1018	80	10,594	78	4.2	−4.9
No	243	19	2806	21		
Missing	10	1	131	1		

Group 7: Mental/physical wellbeing						
Current psychiatric problems						
No problems	1083	85	11,216	83	5.9	−2.5
Some problems	124	10	1371	10	−1.3	−0.4
Significant problems	63	5	909	7	−7.6	4.5
Missing	1		35			
History of psychiatric treatment						
Yes	111	9	1375	10	−4.9	4.9
No	1160	91	12,156	90		
Emotional wellbeing linked to offending behaviour						
Yes	503	40	5537	41	−2.9	−2.9
No	754	59	7823	58		
Missing	14	1	171	1		
Has physical or mental health conditions which need to be taken into account						
Yes	524	41	5777	43	−3.2	−6.0
No	727	57	7506	55		
Missing	20	2	248	2		

Group 8: Offending history from PNC records						
Mean years since last recorded offence	0.35	±0.69	0.45	±0.68	−15.7	−3.3
Mean previous acquisitive crimes						
<4 weeks	0.32	±0.75	0.21	±0.63	17.2	4.5
4wks to 6 months	1.21	±1.71	1.06	±1.68	8.9	1.4
6 months to 1 year	0.89	±1.59	0.90	±1.54	−0.8	−2.1
Over one year	20.7	±22.0	21.6	±21.2	−3.9	−7.8
Previous non-acquisitive crimes						
<4 weeks	0.14	±0.53	0.11	±0.49	5.4	4.5
4 weeks to 6 months	0.65	±1.24	0.64	±1.34	0.8	−0.3
6 months to 1 year	0.52	±1.09	0.63	±1.33	−8.8	1.6
Over one year	13.8	±14.9	14.7	±15.3	−6.0	−6.8
Breach offences						
<4 weeks	0.17	±0.49	0.18	±0.52	−2.1	0.0
> 4weeks	8.60	±8.21	8.73	±7.59	−1.7	−1.0

**Fig. 2:**
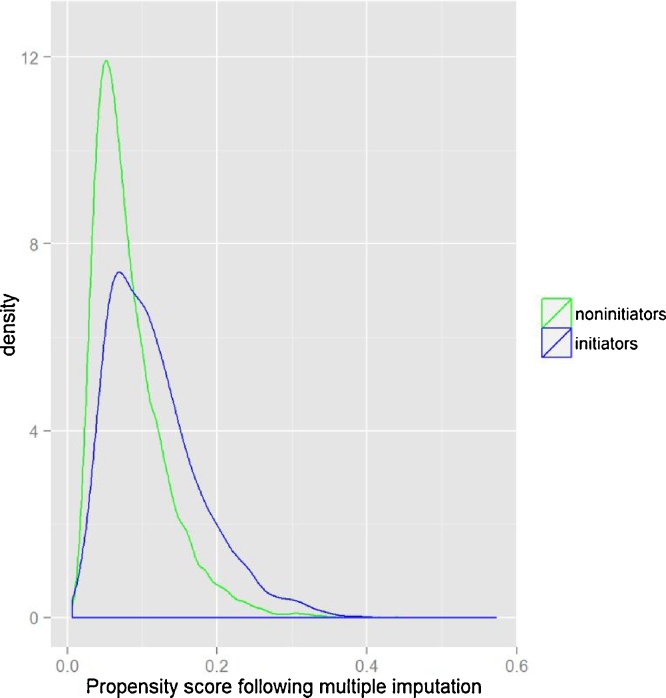
Distribution of propensity scores, averaged across 10 multiple imputation datasets, for initiator and non-initiator assessments where height indicates density of subjects.

Overall there was good overlap of propensity scores between *initiators* and *non-initiators*, implying that there was good availability of potential matches (Fig. 2). Following multiple imputation, to account for missing data, 95% of *initiators* (1201) and 37% of *non-initiators* (5047) were included in the analysis dataset.

After matching, all standardised differences were within the pre-specified 10% threshold, indicating that the propensity score matching approach was successful in achieving covariate balance between *initiators* and *non-initiators* (Table 2). Therefore, further models which included interactions and higher order terms were judged unnecessary.

### Drug-related poisoning risk initiators vs. non-initiators

There were 84 DRPs over follow-up, resulting in a mortality rate of 6.5 DRPs per 1000 person years (95% CI: 5.2–8.0). The rate of DRPs for *initiators* was less than half that for *non-initiators* (Table 3). In the unmatched proportional hazard analysis, there was insufficient evidence to conclude a reduction in DRP following initiator-assessments (p = 0.12). In the matched analyses there was modest evidence of a reduction in DRP deaths (p = 0.06).

**Table 3:** Results of drug-related poisoning mortality analyses, comparing initiators and non-initiators: unmatched, propensity score matching and propensity score matching following multiple imputation (Number of assessments = 14,802).

Analysis	Group	Deaths	Person years	Rate, per 1000 person year [95% CI]	Hazard ratio [95% CI]	p-value
Unmatched	Initiators	3	1104	2.7 [0.9, 8.4]	0.40 [0.13, 1.26]	0.116
	Non-initiators	81	11,844	6.8 [5.5, 8.5]	Ref	

1:5 matched	Initiators	2	780	2.6 [0.6, 10.2]	0.30 [0.09, 0.99]	0.048
	Non-initiators	25	3251	7.7 [5.2, 11.4]	Ref	

1:5 matched after MI	Initiators	3	1049	2.9 [0.9, 8.9]	0.42 [0.17, 1.04]	0.060
	Non-initiators	30	4413	6.8 [4.8, 8.9]	Ref	

### Acquisitive offending risk initiators vs. non-initiators

The overall rate of recorded acquisitive offences was 0.91 per person year (95% CI: 0.86–0.96). The risk of an acquisitive offence was 22% higher following *initiator a*ssessments than following *non-initiator* assessments (Table 4). In the matched analysis, the difference narrowed to 8%, and then 10% after multiple imputation.

**Table 4:** Results of acquisitive crime analyses, comparing initiators and non-initiators: unmatched, propensity score matching and propensity score matching following multiple imputation (No of assessments = 14,802).

Analysis	Group	Crimes	Person years	Rate, per person year [95% CI]	Hazard ratio [95% CI]	p-value
Unmatched	Initiator	544	666	0.82 [0.75, 0.89]	1.22 [1.11, 1.33]	<0.001
	Non-initiators	5261	7965	0.66 [0.64, 0.68]	Ref	

1:5 matched	Initiators	391	464	0.84 [0.76, 0.93]	1.08 [0.99, 1.17]	0.102
	Non-initiators	1579	2079	0.76 [0.72, 0.80]	Ref	

1:5 matched after MI	Initiators	513	636	0.81 [0.74, 0.88]	1.10 [1.02, 1.18]	0.015
	Non-initiators	2052	2860	0.72 [0.69, 0.75]	Ref	

### Sensitivity analyses

Changing the length of the classification phase from two weeks to one week or four weeks did not substantially change the estimates with respect to DRPs or acquisitive offences, although the p-values for the DRP analysis got larger (Appendix C in Supplementary material).

## Discussion

There was a modestly significant reduction in DRP deaths associated with *being an initiator* that is consistent with the hypothesis that initial treatment contact is effective in reducing DRP deaths. However, the current study, in isolation, cannot establish this due to a lack of statistical power, as indicated by the wide confidence intervals. In contrast, the study found that, after adjusting for observed confounding, *initiators* had a ten percent higher offending rate, with lower 95% confidence interval close to neutrality. This result is at odds with the hypothesis that treatment contact is effective at reducing offending for offending-heroin users.

The study was designed to emulate elements of a controlled trial: using eligibility criteria; balancing groups at baseline; and analysing groups as treatment was initially planned, regardless of future treatment status. A large proportion of assessments were excluded from the analysis, mostly because, at the time of their assessment, the subject was already in treatment or they were incarcerated. These exclusion criteria were purposefully established, prior to analysis, to focus the study on the question: for heroin users identified from community probation, what is the effect of initiating contact with drug treatment services on future DRP mortality/acquisitive offending? Adopting eligibility criteria matches what occurs in randomised trials and has been recommended for wider use in observational research (Danaei et al., 2013, Hernan et al., 2008).

Diagnostics showed that propensity score matching provided a cohort where the *initiator* and *non-initiator* assessments were balanced on observed covariates. However, we cannot rule out residual confounding because either: (i) there are further unobserved variables which explain group differences and are also related to outcome; or (ii) the covariates were poorly measured so that they failed sufficiently to capture the true difference.

Although an extensive set of covariates were available from probation assessments, in addition to historical offending information from police records, bias by unobserved confounding cannot be ruled out. The probation interview is specifically designed to assess future offending likelihood and has previously shown utility in this regard, (Howard, 2011, Howard and Moore, 2009) however they might be less effective at identifying drug treatment need. Given that most covariates came from self-report, disclosure bias may mask true differences between groups. One study of the inter-rater reliability of probation interviews concluded that questions relating to drug use and accommodation had good reliability, whilst those relating to prior alcohol use and risk of serious harm had poor reliability (Morton, 2009). In England and Wales, when the cause of death is deemed to be unnatural then it is subject to a coroner inquest. Coding of the cause of death from a coroner’s report is subject to error, and there may be some familial pressure to code the death as something other than drug-related, resulting in an underreporting of the number of DRPs.

One reason to believe that residual confounding exists is that there is no compelling theory to support an increase in offending following treatment contact, albeit the small one observed here. There is some confounding evident in the analysis: the estimated increase in offending, associated with being an initiator, changed from twenty to eight percent after propensity-score matching. The amount of unobserved confounding would have to be greater than that observed to change the inference towards a positive effect of treatment contact and given the number and range of variables used to calculate propensity scores, this seems unlikely. It should be noted that a recent UK-based randomised trial (Holland et al., 2014) found a similarly counter-intuitive result: supervised consumption of methadone over three months, compared to one-week supervision, resulted in an increase in offending (OR = 3.37, 95% CI: 1.3–8.9). In conjunction with this, our finding may warrant fresh investigation into the relationship between treatment and offending.

Utilising records from probation assessments allows subjects to be identified prior to entering treatment and provides an extensive set of variables to achieve balance. This approach has previously been used to assess the effect of alcohol treatment referral on recidivism — a study that found no overall impact of the intervention (McSweeney, 2014). Alternative observational study designs compare treated and untreated periods. The DRP-risk reduction estimated here was consistent with that seen in such studies (Cornish, Macleod, Strang, Vickerman, & Hickman, 2010; Pierce, Bird et al., 2016; Sordo et al., 2017). In contrast to the findings presented here, studies of this type that investigate offending outcomes have tended to show lower rates in treatment than those observed out of treatment (Bukten et al., 2011; Lind, Chen, Weatherburn, & Mattick, 2005).

One difference between these studies and the current one is the different measures of treatment effectiveness. The object of the current analysis is to estimate the effect of *initial contact* with treatment, whilst other observational studies tend to estimate the effect of *receiving* treatment. The effect of initiating treatment is also likely to be more conservative because addiction-treatment tends to be most effective whilst subjects are being treated. To illustrate, in one Australian study of offending among prison leavers, comparing those who were on methadone maintenance at baseline with those who were not resulted in a Hazard ratio of 0.98 (95% CI: 0.88–1.09) and analysing according to being in treatment resulted in an HR of 0.80 (95% CI: 0.71–0.90) (Larney, Toson, Burns, & Dolan, 2012). One difficulty in measuring the effect of receiving treatment is that, because treatment exposure changes over follow-up, time-updated covariates are needed to account for confounding, but in most cases such variables will not be available. Even if sufficient data were collected, when treatment affects the future values of a time-dependent confounder standard estimation methods will still produce biased estimates (Pierce, Dunn, & Millar, 2016). Regardless, the effect of initial treatment contact is of greater interest when assessing the effectiveness of current policy, as is the aim of the present analysis.

Another reason why our study might fail to reveal an effect of treatment on offending is because the cohort is restricted to those who were identified in the criminal justice system, with 63% of *initiators* have been referred to treatment from within the criminal justice system (compared to the national proportion of 26%) (National Treatment Agency for Substance Misuse, 2011). The lack of evidence for criminal justice referral as a method of accessing treatment has been identified in a recent systematic review (Hayhurst et al., 2015).

The results presented may be due to poor treatment adherence amongst those who started treatment and the negative effects of leaving treatment. It is noted that 6.4% of initiators (81/1271) did not receive any treatment following their triage appointment and we cannot expect treatment contact to be effective for these. A future analysis could use a similar design to investigate the effect of discontinuing treatment, selecting patients in long-term treatment and comparing those who discontinue treatment with those who do not. Irrespective of the lack of an effect on crime, it is striking that a very small proportion of those identified at a formal OASys assessment interview as regular heroin users, for whom treatment would appear to be indicated, subsequently went on to seek treatment.

The findings reported here raise questions concerning the referral of drug using offenders for treatment as a means to reduce crime, insofar as *initiation of contact* was not associated with a lower risk of offending among these CJS-involved participants. However, if the effect on DRP-deaths was established with further good-quality evidence, this could provide sufficient justification for continued investment, especially given the record-high number of DRP-deaths in the most recent statistics published in England and Wales (Office for National Statistics, 2016). It has been previously noted that the criminal justice system has lagged behind medical research in adopting RCTs (Bird, Goldacre, & Strang, 2011) and this ought to be addressed if we are to improve outcomes for offenders.

## Declarations of interest

**Millar** has received research funding from the UK National Treatment Agency for Substance Misuse and the Home Office. He has been a member of the organising committee for conferences supported by unrestricted educational grants from Reckitt Benckiser, Lundbeck, Martindale Pharma, and Britannia Pharmaceuticals Ltd, for which he received no personal remuneration. He is a member of the Advisory Council on the Misuse of Drugs.

**Bird** holds GSK shares. She is formerly an MRC programme leader and has been elected to Honorary Professorship at Edinburgh University. She chaired Home Office’s Surveys, Design and Statistics Subcommittee (SDSSC) when SDSSC published its report on 21st Century Drugs and Statistical Science. She has previously served as UK representative on the Scientific Committee for European Monitoring Centre for Drugs and Drug Addiction. She is co-principal investigator for MRC-funded, prison-based N-ALIVE pilot Trial.

**Marsden** works in an integrated university (Institute of Psychiatry, Psychology and Neuroscience [IOPPN], King’s College London) and National Health Service Academic Health Sciences Centre (King’s Health Partners) and declares the following financial relationships: in addition to university-based addiction treatment-related research grants from the Department of Health, Institute for Health Research (Health Technology Assessment programme), and the Biomedical Research Centre for Mental Health (South London and Maudsley NHS Foundation Trust and IOPPN), he has part-time employment as Senior Academic Advisor for the Alcohol, Drugs and Tobacco Division, Health and Wellbeing Directorate, Public Health England; educational grant funding at King’s College London via Reckitt Benckiser Pharmaceuticals (RBP) to Action on Addiction for a study of psychological interventions in opioid dependence (2010–2016); consultation to RBP (2011) and Merck Serono (2013), and honoraria as co-chair of the Improving Outcomes in Treatment of Opioid Dependence conference (2015) via educational grant funding from RBP [Indivior PLC] to PCM Scientific. He holds no stocks in any company.

**Seddon** has received research funding from the UK National Treatment Agency for Substance Misuse and the Home Office.

No further declarations
