# Supplementary material for: Effect of initiating drug treatment on the risk of drug-related poisoning death and acquisitive crime among offending heroin users
Source: Int J Drug Policy. 2018 Jan;51:42–51. doi: 10.1016/j.drugpo.2017.09.017 (PMC5788328; doi:10.1016/j.drugpo.2017.09.017)
Supplement: Supplementary file 1 [file mmc1.docx]

**APPENDICES: Effect of initiating drug treatment on the risk of drug-related poisoning death and acquisitive crime among a cohort of offending heroin users**

**Appendix A** Description of variables used in the analysis

**Group 1: demographic variables**

| **Variable** | **Derived?** | **Description** |
| --- | --- | --- |
| gender | No | Gender |
| age | No | Age at assessment |
| White | Yes | White ethnicity |
| region | Yes | Region in the UK: Eastern; East Midlands; London; North East; North West; South East; South West; West Midlands; Yorkshire and Humber. |

**Group 2: relating to the assessment**

| **Variable** | **Derived?** | **Description** |
| --- | --- | --- |
| purposegroups | Yes | Purpose of the assessment: Pre-sentence report; Start community sentence; sentence plan review; pre-release; start licence; End sentence |
| sentencegroup | Yes | sentence type tied to the assessment: Community sentence (CJA 03); Suspended sentence; Other; Community punishment/rehabilitation/punishment and rehabilitation order. |
| ass_year | Yes | Financial year in which the assessment took place |
| ass_no | Yes | Assessment number included in analysis dataset |

**Group 3: Offending which led to assessment**

| **Variable** | **Derived?** | **Description** |
| --- | --- | --- |
| offence | Yes | offence which resulted in assessment: Violent or sexual; serious acquisitive crime; non-serious acquisitive crime; drugs offences; other |
| s2q13_escalation_seriousness | No | Are current offence(s) an escalation in seriousness from previous offending |
| s2q14_established_pattern | No | Are current offence(s) an established pattern of similar offending? |

**Group 4: Drug use/problems**

| **Variable** | **Derived?** | **Description** |
| --- | --- | --- |
| s2q9_addiction | No | Was there evidence of motivation due to addictions/perceived needs |
| s2q10_drugs | No | Did drugs act as a disinhibitor? |
| s8q1a_frequency | No | Weekly or daily heroin use |
| s8q1a_drug_injected | No | heroin injected |
| s8q1a_previous_drug_injected | No | Previously inejcted heroin |
| other_opiates | Yes | Other opiates or (non-prescribed) methadone |
| crack | Yes | Crack use, grouped: not used; occasionally (or monthly); weekly or daily |
| s8q1d_previous_use | No | previous (but not current) crack use |
| benzos | Yes | Use of benzodiazipines, grouped: not used; occasionally (or monthly); weekly or daily |
| co_amph | Yes | Use of cocaine or amphetamines, weekly or more frequent |
| other | Yes | Use weekly or more frequent of prescribed, hallucinogens, ecstasy, steroids, or other |
| injected | Yes | a current injector of a drug which isn't heroin |
| s8q7_violent_behaviour | No | violent behaviour related to dryug use |
| s8q8_motivation_tackle_misuse | No | Motivatad to tackle drug misuse? (Evidence of problems recognised? Consider whether s/he recognises or is motivated to reduce drug dependency and wheteher s/he is capable of change and wants to/has attended treatments/ programmes) |
| s8q9_drug_use_main_activity | No | drug use and obtaining drugs a major activity/occupation |
| s8_linked_to_risks | No | Drug s misuse issues linked to risk of serious harmn, risks to the individual and other risks |
| s8_linked_to_behavior | No | Drugs misuse issues linked to offending behaviour |

**Group 5: Alcohol use**

| **Variable** | **Derived?** | **Description** |
| --- | --- | --- |
| s2q10_alcohol | No | Did alcohol act as a disinhibitor? |
| s9q1_current_use | No | Is current alcohol use a problem |
| s9q2_binge_drinking | No | Binge drinking or excessive use of alcohol in the last 6 months |
| s9q3_past_misuse | No | Frequency and level of alcohol misuse in the past |
| s9q5_motivation_tackle_misuse | No | Motivated to tackle alcohol misuse? |
| s9_linked_to_behavior | No | Alcohol misuse linked to offending behaviour |

**Group 6: Employment/housing**

| **Variable** | **Derived?** | **Description** |
| --- | --- | --- |
| unstable_accom | Yes | Is he/she in unstable accommodation |
| unemployed | Yes | Is he/she unemployed |
| benefits | Yes | Is he/she in receipt of benefits |
| s5_linked_to_behavior | No | Are the financial issues linked to offending behaviour |

**Group 7: Mental/physical wellbeing**

| **Variable** | **Derived?** | **Description** |
| --- | --- | --- |
| s10q6_psychiatric_problems | No | current psychiatric problems |
| s10q7_psychiatric_treatment | No | History of psychiatric treatment |
| s10_linked_to_behavior | No | Emotional well being linked to offending behaviour |
| s13q1_general_health | No | Does the offender have any physical or mental health conditions which need to be taken into account? |

**Group 8: Offending history from PNC records**

| **Variable** | **Derived?** | **Description** |
| --- | --- | --- |
| last_offence | Y | Time since last recorded offence in PNC |
| _4wks_acq | Y | Acquisitive offences in the past 4 weeks |
| _6mnth_acq | Y | Acquisitive offences: 4wks up to 6 months |
| _1yr_acq | Y | Acquisitive offences: 6 months up to 1 year |
| older_acq | Y | Acquisitive offence over 1 year |
| _4wks_nonacq | Y | Non-acquisitive offences, excluding breach offences in the past 4 weeks |
| _6mnth_nonacq | Y | Non-acquisitive offences, excluding breach offences: 4wks up to 6 months |
| _1yr_nonacq | Y | Non-acquisitive offences, excluding breach offences: 6 months up to 1 year |
| older_nonacq | Y | Non-acquisitive offence over 1 year |
| _4wks_breach | Y | Breach offences in the past 4 weeks |
| older_breach | Y | Breach offences over 4 weeks |

**Appendix B: Algorithm used in propensity score matching**

1. For each initiators, identify all non-initiators within the pre-defined calliper distance, set as 20% of the standard deviation of the logit of the propensity scores[1]
2. Sort the list of all (one to many) initiator to non-initiator matches, ordered by propensity score distance from smallest to greatest, sorting ties randomly
3. For each non-initiator in the list, move the match with the smallest propensity score distance to an analysis dataset.
4. For each initiator, keep up to 5 non-initiator matches.

[1] Austin PC: **Optimal caliper widths for propensity-score matching when estimating differences in means and differences in proportions in observational studies.** *Pharm Stat* , **10**:150–61.

**Appendix C Sensitivity analyses**

| **Analysis** | **Group** | **No assessments** | **N** | **Rate, per 1,000 person year [95% CI]** | **Hazard ratio [95% CI]** | **p** |
| --- | --- | --- | --- | --- | --- | --- |
| Sensitivity 1: cut-off 1 week (DRP) | Initiators | 839 | 3 | 4.1 [1.3, 12.8] | 0.58 [0.18, 1.84] | 0.358 |
|  | Non-initiators | 13,970 | 87 | 7.1 [5.7, 8.7] | Ref |  |
|  |  |  |  |  |  |  |
| Sensitivity 2: cut-off 4 weeks (DRP) | Initiators | 1,944 | 8 | 4.8 [2.4, 9.6] | 0.74 [0.36, 1.54] | 0.425 |
|  | Non-initiators | 12,852 | 72 | 6.5 [5.1, 8.1] | Ref |  |
|  |  |  |  |  |  |  |
| Sensitivity 1: cut-off 1 week (acquisitive offence) | Initiators | 839 | 372 | 0.83 [0.75, 0.92] | 1.19 [1.07, 1.32] | 0.001 |
|  | Non-initiators | 13,970 | 5,750 | 0.69 [0.67, 0.71] | Ref |  |
|  |  |  |  |  |  |  |
| Sensitivity 2: cut-off 4 weeks (acquisitive offence) | Initiators | 1,944 | 741 | 0.76 [0.70, 0.81] | 1.22 [1.13, 1.32] | <0.001 |
|  | Non-initiators | 12,852 | 4,469 | 0.61 [0.59, 0.63] | Ref |  |
|  |  |  |  |  |  |  |
